# Identifying the causal relationship between sedentary behavior and heart failure: Insights from a Mendelian randomization study and mediation analysis

**DOI:** 10.1002/clc.24101

**Published:** 2023-08-29

**Authors:** Xifeng Zheng, Manqi Liu, Zijun Wu, Zhen Jia

**Affiliations:** ^1^ Department of Cardiology Affiliated Hospital of Guangdong Medical University Zhanjiang Guangdong China; ^2^ Department of Geriatrics Affiliated Hospital of Guangdong Medical University Zhanjiang Guangdong China

**Keywords:** heart failure, inverse variance weighting model, mediation analysis, Mendelian randomization study, sedentary behavior

## Abstract

**Background:**

Observational studies have revealed that a lack of physical exercise may be linked to a higher risk of heart failure (HF). Here, the causal relationship between sedentary behavior (SB) and HF was investigated using Mendelian randomization (MR).

**Hypothesis:**

SB was considered as an important risk factor of HF.

**Methods:**

Single nucleotide polymorphisms with a genome‐wide statistical significance threshold of <5 × 10^−8^ among the SB‐proxied phenotypes (TV screen time, computer use, and driving) from genome‐wide association study (GWAS) datasets were identified as instrumental variables (IVs). The MR study was performed using the inverse‐variance weighting (IVW) model as a primary standard to evaluate causal relationships. Simultaneously, MR‐Egger regression, weighted median, and maximum likelihood models were used as supplements. Sensitivity analysis, consisting of a heterogeneity and horizontal pleiotropy test, was performed using Cochran's Q, MR‐Egger intercept, and MR‐PRESSO tests to ensure the reliability of conclusions.

**Results:**

The IVW model results showed that increased TV screen time correlated with a higher genetic susceptibility for HF in both HF‐associated GWAS datasets, which was also supported by weighted median and maximum likelihood model results. The odds ratios with 95% confidence intervals were 1.418 (1.182–1.700) and 1.486 (1.136–1.943), respectively. Although the results of Cochran's *Q* test indicated certain heterogeneity among the IVs. The MR‐Egger intercept and MR‐PRESSO tests suggested no horizontal pleiotropy and verified the reliability of the conclusion.

**Conclusions:**

This MR study identified that increased TV screen time may predispose individuals to the development of HF.

## Graphical abstract

This Mendelian randomization study identified that increased TV screen time may predispose individuals to the development of heart failure (HF). Meanwhile, coronary artery disease, hypertension, type 2 diabetes, obesity, and body mass index were considered as major mediators in how sedentary behavior results in or aggravates HF.

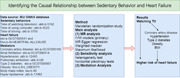

**Abbreviations**
CI: confidence interval
GWAS: genome‐wide association study
HF: heart failure
IV: instrumental variable
IVW: inverse variance weighting
MR: Mendelian randomization
OR: odds ratio
SB: sedentary behavior
SNP: single‐nucleotide polymorphism

## 1 INTRODUCTION

Heart failure (HF) is a complex clinical syndrome with symptoms and signs resulting from any structural or functional impairment of ventricular filling or ejection of blood from the heart. This condition affects more than 26 million people worldwide and is considered a major cause of mortality and poor quality of life. The prevalence of HF continues to increase with aging populations and creates a significant economic burden on the public health system.^1^, ^2^ Exploration and identification of controllable lifestyle‐associated risk factors are essential for HF management and prevention. Accumulating epidemiological evidence suggests that sedentary behavior (SB) is associated with increased morbidity of cardiovascular diseases and HF.^3^, ^4^ SB is a lifestyle where physical exercise is lacking. The coronavirus disease 19 (COVID‐19) pandemic may have increased the prevalence of SB. There is no consensus regarding the definition of SB; however, the most representative definition refers to any conscious behavior characterized by an energy consumption of ≤1.5 metabolic equivalents in seated or reclined positions.^5^, ^6^, ^7^ Zhao et al.^8^ published a meta‐analysis reporting that SB is associated with increased all‐cause mortality and decreased quality of life in patients with HF. Nevertheless, to the best of our knowledge, there is limited evidence of the causal relationship between SB and HF, and a critical lack of large‐sample cohort studies.

Mendelian randomization (MR) is a method that uses valuable genetic variants as instrumental variables (IVs) to evaluate the causal effects between nongenetic and modifiable risk factors and diseases. In the absence of randomized controlled trials (RCTs), MR studies represent an alternative strategy for obtaining causal inference since genetic variants are randomly assigned during meiosis, simulating the conditions of RCT. Compared with traditional observational studies, MR is less likely to be influenced by unmeasured confounding factors since genetic variants are identified at the time of conception.^9^, ^10^ This technique has been applied successfully to a range of causal relationship studies between behavioral exposures and cardiovascular diseases.^11^, ^12^, ^13^ This study aimed to identify the causal relationship between SB and HF using MR based on one million samples cohort from the European population taken from publicly available genome‐wide association study (GWAS) datasets.

## 2 MATERIALS AND METHODS

### 2.1 Study design and information on SB‐ and HF‐associated GWAS datasets

This MR study depended on three fundamental assumptions to achieve impartial results. Firstly, the selected genetic IVs must be significantly associated with the exposure factor. The IVs should also be independent of potential confounders associated with exposure factors and outcomes and should affect the outcomes only through the exposure factor.^9^

The requirement for ethical committee approval in this study was waived because the genetic variable information of single nucleotide polymorphisms (SNPs) was obtained from the IEU GWAS database (https://gwas.mrcieu.ac.uk/datasets/), a publicly available GWAS summary database.^14^ To obtain more comprehensive conclusions on the causal relationship between SB (exposure) and HF (outcome), datasets with the largest sample size and number of SNPs sequenced were selected. SB was proxied by three behavioral exposures (time spent watching television, using a computer, and driving) that were derived from the UK Biobank database.^15^ Two HF‐associated GWAS datasets were derived from the Heart Failure Molecular Epidemiology for Therapeutic Targets (HERMES) Consortium^16^ and FinnGen biobank.^17^ All GWAS datasets involved in this study included populations of European ancestry to mitigate bias from population stratification. Detailed information on the datasets used in the study is listed in Table 1.

**Table 1:** Basic information about the datasets used in the study.

Traits	GWAS ID	Years	Population	Sample size
Sedentary behaviors				Total sample
Time of watching television	ukb‐b‐5192	2018	European	437 887
Time of using computer	ukb‐b‐4522	2018	European	360 895
Time of driving	ukb‐b‐3793	2018	European	310 555
Outcome				Case/control
Heart failure	ebi‐a‐GCST009541	2020	European	47 309/930 014
	finn‐HEARTFAIL*	2021	European	23 397/194 811
Mediators				Case/control
Coronary artery disease	ebi‐a‐GCST005195	2017	European	122 733/424 528
Atrial fibrillation	ebi‐a‐GCST006414	2018	European	60 620/970 216
Hypertension	ukb‐b‐12493	2018	European	54 358/408 652
Pulmonary embolism	ukb‐d‐I26	2018	European	2 118/359 076
Type 2 diabetes	ebi‐a‐GCST006867	2018	European	61 714/593 952
Obesity	finn‐b‐E4_OBESITY	2021	European	8908/209 827
Body mass index	ieu‐b‐40	2018	European	681 275/2 336 260
Hyperlipidaemia	ukb‐b‐17462	2018	European	3439/459 571

*Note*: finn‐HEARTFAIL is short for finn‐b‐I9‐HEARTFAIL‐ALLCAUSE.

Abbreviation: GWAS, genome‐wide association study.

### 2.2 Selection criteria for SNPs used as IVs

SNPs used as IVs must pass muster by meeting a genome‐wide statistical significance threshold (<5 × 10^−8^) among SB‐proxied phenotypes in GWAS datasets. They also needed to have irrelevant relationships with confounders and have affected the outcome only through the exposure.^18^ The corresponding linkage disequilibrium was tested to confirm the presence of SNPs and that these SNPs were independent; this was done by trimming SNPs within a 10 000 kb window at a threshold of *r* ^2^ < .001. *F*‐statistics (>10) were used to evaluate the strength of the IVs to avoid the influence of weak instrumental bias. The SNPs used as IVs were further matched with those in HF‐associated GWAS datasets to establish genetic associations. The summary SNP‐phenotype and SNP‐outcome statistics were then harmonized to ensure effect size alignment and palindromic SNPs were excluded.^19^

### 2.3 MR study and sensitivity analysis

The MR study was performed using a random effect inverse‐variance weighting (IVW) model as the primary standard,^20^ and three other methods (MR‐Egger regression,^21^ weighted median,^22^ and maximum likelihood^23^) were used as supplements to evaluate the potential causal relationship between SB and HF under various scenarios. Sensitivity analysis was performed to measure the reliability and stability of the model conclusion, consisting of Cochran's *Q* test (heterogeneity was identified when *p* < .05 according to the IVW model or MR‐Egger regression), horizontal pleiotropy test using the MR‐Egger intercept^24^ and MR‐PRESSO test,^25^ and a “leave‐one‐out” test (each SNP was abandoned successively and the IVW analysis was repeated to identify whether the causal relationship estimate was driven by a certain SNP). The results were reported as odds ratios (ORs) with corresponding 95% confidence intervals (CIs) and *p*‐values and were illustrated as scatter plots and funnel plots. The R 4.0.3 software with “TwoSampleMR”^26^ and “MR‐PRESSO”^25^ packages was used to process data and visualize the results.

### 2.4 Mediation analysis

Mediational analysis is a methodology for disaggregating the impact of exposure (SB) on outcomes (HF) that act directly and through mediating variables. The effects are decomposed by using multivariate analysis to estimate the causal relationships among three types of variables: exposure, mediator, and outcome.^27^ Mediation analysis was conducted using the IVW model to investigate the mediating effects of primary cardiac diseases (coronary artery disease, hypertension, atrial fibrillation, and pulmonary embolism) and metabolic‐related disorders (type 2 diabetes, hyperlipidemia, obesity, and body mass index) on how SB results in or aggravates HF. The illustration of mediation analysis as below.


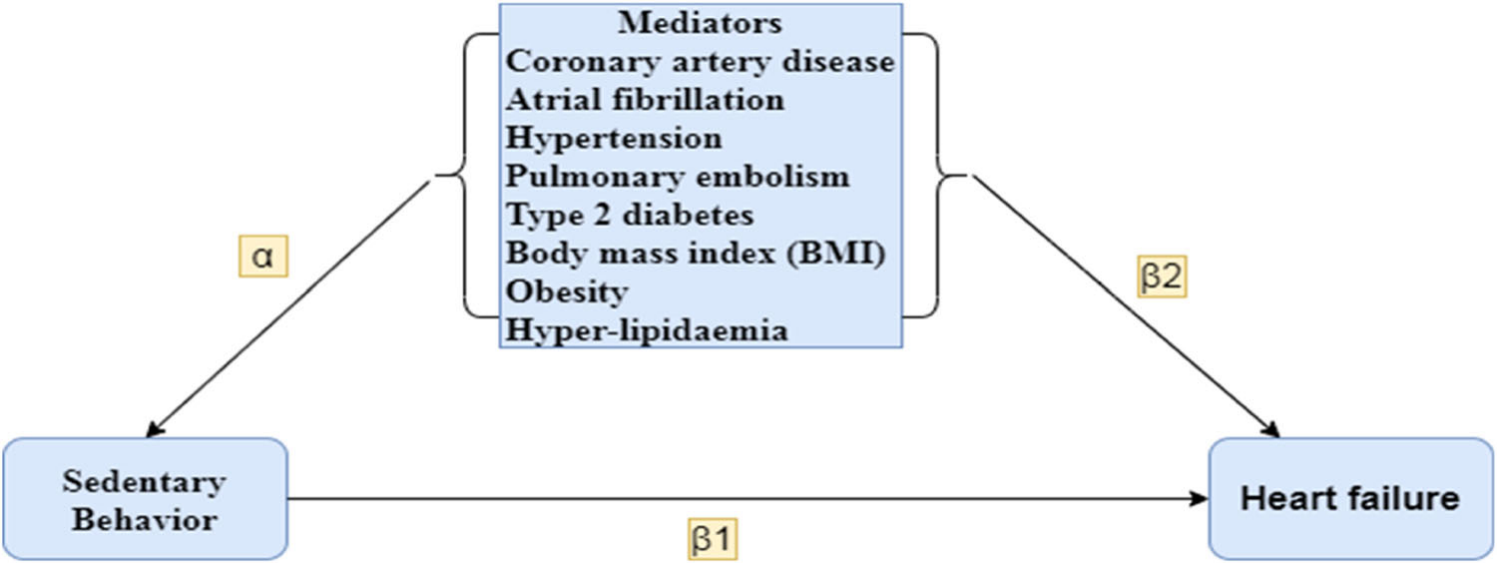


*Note*: Illustration of the direct and mediation effects of SB on HF. *β*1 represents the direct effect of SB on HF. *αβ*2 represents the mediating effect that SB on HF through the mediators included in the model. The total effect represents *β*1 + *αβ*2.

## 3 RESULTS

### 3.1 IVs identified and results of MR

A total of 100, 75, and 6 SNPs were ultimately identified as IVs from different SB‐proxied phenotypes in HF‐association GWAS ebi‐a‐GCST009541 datasets for TV screen time, computer screen time, and driving, respectively; 105, 76, and 6 were identified, respectively, in HF‐association GWAS finn‐HEARTFAIL datasets. The *F*‐statistic scores of all selected SNPs were more than 10, indicating a low risk of weak‐instrument bias.

**Figure 1:**
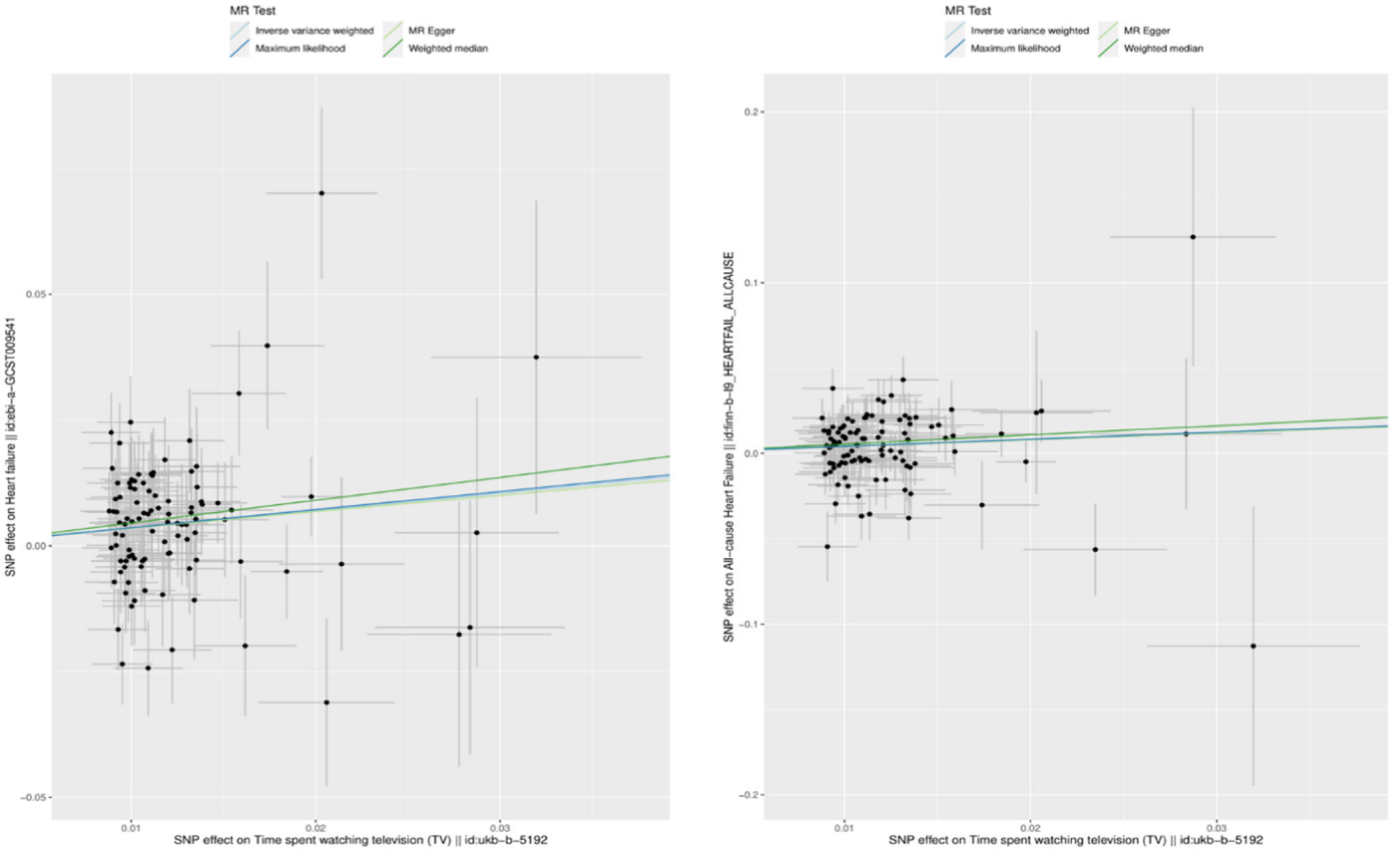
Scatter plots of the Mendelian randomization (MR) study results.

*Note*: Each scatter plot point is an IV SNP. Each diagonal line in a different color is a testing model. The left plot represents the relationship of “TV screen time” and HF in the GWAS data set ebi‐a‐GCST009541, and the right represents the relationship of “TV screen time” and HF in the GWAS data set finn‐HEARTFAIL.

Taking the result of the random effect IVW model as the primary standard, a causal relationship between the phenotype of “TV screen time” and HF could be inferred in both HF‐associated GWAS datasets (ebi‐a‐GCST009541: OR = 1.418, 95% CI = 1.182–1.700, *p* < .001, finn‐HEARTFAIL: OR = 1.486, 95% CI = 1.136–1.943, *p* = .003). This conclusion was also supported by the weighted median model and maximum likelihood model. The results from the MR‐Egger regression model did not exhibit significant statistical differences. Despite this, the value of the effect was consistent with that of the other three models, supporting the conclusion that watching TV excessively can lead to a higher probability of HF. No causal relationship with HF was detected between the genetic liability for the other two phenotypes (computer screen time and driving). Detailed information of MR study is displayed in Table 2 and is illustrated as a scatter plot (Figure 1).

**Table 2:** Results of the Mendelian randomization study.

	Heart failure (ebi‐a‐GCST009541)				Heart failure (finn‐HEARTFAIL)			
	SNPs (*n*)	*β*	OR (95% CI)	*p*‐Value	SNPs (*n*)	*β*	OR (95% CI)	*p*‐Value
Time spent watching television								
Inverse variance weighted	100	.349	1.418 (1.182–1.700)	<.001*	105	.396	1.486 (1.136–1.943)	.003*
MR‐Egger regression	100	.326	1.386 (0.605–3.176)	.442	105	.393	1.482 (0.394–5.578)	.562
Weighted median	100	.453	1.574 (1.252–1.978)	<.001*	105	.539	1.715 (1.234–2.384)	.001*
Maximum likelihood	100	.360	1.433 (1.228–1.673)	<.001*	105	.412	1.509 (1.216–1.874)	<.001*
Time spent using computer								
Inverse variance weighted	75	−.045	0.956 (0.800–1.142)	.619	76	−.186	0.831 (0.629–1.097)	.191
MR‐Egger regression	75	−.526	0.591 (0.238–1.467)	.261	76	−.200	0.819 (0.171–3.930)	.803
Weighted median	75	−.033	0.968 (0.763–1.226)	.785	76	−.119	0.887 (0.621–1.268)	.511
Maximum likelihood	75	−.046	0.955 (0.812–1.122)	.577	76	−.193	0.824 (0.654–1.038)	.100
Time spent driving								
Inverse variance weighted	6	.627	1.872 (1.046–3.349)	.035*	6	.338	1.402 (0.616–3.193)	.420
MR‐Egger regression	6	−1.677	0.187 (0.001–45.021)	.581	6	−2.168	0.114 (0.001–320.04)	.621
Weighted median	6	.451	1.570 (0.754–3.270)	.229	6	.364	1.440 (0.521–3.977)	.482
Maximum likelihood	6	.639	1.895 (1.046–3.432)	.035*	6	.346	1.413 (0.617–3.239)	.413

*Note*: SNPs means number of SNP as instrumental variables. *β* Means effect value.

Abbreviations: CI, confidence interval; MR, Mendelian randomization; OR, odds ratio; SNP, single nucleotide polymorphism.

### 3.2 Results of sensitivity analysis

The result of Cochran's *Q* test using the IVW model and MR‐Egger regression suggested certain heterogeneity (*p* < .05) among the IVs in the TV screen time GWAS data set. This limitation has no fundamental impact on the conclusion that a causal relationship exists between the TV‐watching phenotype and HF as drawn by the random effect IVW model. More importantly, both the results of MR‐Egger intercept and MR‐PRESSO test demonstrated that the IV of TV screen time passed the horizontal multiplicity test (Table 3). In addition, the results of the “leave‐one‐out” method (Supporting Information: Figure S1) indicated that no specific SNP had a significant effect on the overall result. Taken together, the results of the sensitivity analysis verified the robustness of the conclusions.

**Table 3:** The results of the heterogeneity and horizontal pleiotropy tests.

Behavior exposures	Heart failure GWAS data set	Heterogeneity test (Cochran Q test)		Horizontal pleiotropy test	
		MR‐Egger regression	IVW model	MR‐Egger intercept	MR‐PRESSO test
TV screen time	ebi‐a‐GCST009541	0.002	0.002	0.956	0.972
	finn‐HEARTFAIL	<0.001	<0.001	0.997	0.785
Using a computer	ebi‐a‐GCST009541	0.073	0.070	0.294	0.252
	finn‐HEARTFAIL	0.002	0.002	0.985	0.788
Driving	ebi‐a‐GCST009541	0.495	0.539	0.454	0.657
	finn‐HEARTFAIL	0.590	0.670	0.567	0.719

*Note*: *p* < .05 was considered as with statistical differences in both heterogeneity test and horizontal pleiotropy test.

Abbreviations: GWAS, genome‐wide association study; IVW, inverse‐variance weighting; MR, Mendelian randomization.

### 3.3 Results of mediation analysis

Coronary artery disease (mediation effect = 0.152, 95% CI = 0.094–0.213, mediated proportion = 43.55%), hypertension (mediation effect = 0.255, 95% CI = 0.179–0.339, mediated proportion = 73.07%), type 2 diabetes (mediation effect = 0.050, 95% CI = 0.020–0.085, mediated proportion = 14.32%), body mass index (mediation effect = 0.271, 95% CI = 0.174–0.379, mediated proportion = 77.65%) and obesity (mediation effect = 0.243, 95% CI = 0.133–0.374, mediated proportion =  69.62%), were identified as major mediators in how SB results in or aggravates HF (Table 4).

**Table 4:** The results of the mediation analysis.

Mediator	Mediation effect (*β* (95% CI))	Proportion of mediation effect
Coronary artery disease	0.152 (0.094–0.213)	43.55%
Atrial fibrillation	0.027 (−0.010–0.063)	–
Hypertension	0.255 (0.179–0.339)	73.07%
Pulmonary embolism	0.0003 (−0.001–0.002)	–
Type 2 diabetes	0.050 (0.020–0.085)	14.32%
Body mass index	0.271 (0.174–0.379)	77.65%
Obesity	0.243 (0.133–0.374)	69.62%
Hyperlipidaemia	0.004 (−0.097–0.109)	–

*Note*: Mediation effect means the effect value of each mediator on the outcome. *β* Means the effect value. The proportion of the mediated effect can be calculated as the “mediated effect/total effect.” Total effect (*β*, 95% CI) = 0.349 (0.167–0.531).

Abbreviation: CI, confidence interval.

## 4 DISCUSSION

Whether due to a draw to an interesting TV show, needing to do work on a computer, or needing to drive, SB is common in daily life and represents a significant proportion of time spent in modern lifestyles. Accumulating epidemiological evidence indicates that SB is a modifiable but significant risk factor associated with the prognosis of HF. Kim et al.^28^ summarized 7 years of data from the National Health and Nutrition Examination Survey database and demonstrated that SB may increase all‐cause mortality in patients with HF. Similarly, a cohort study by LaMonte et al.^29^ focused on postmenopausal women (80 982 samples) with a 9‐year follow‐up concluded that SB was associated with an increased risk of HF hospitalization. Both the 2018 Physical Activity Guidelines for Americans and the World Health Organization 2020 guidelines on physical activity and SB emphasize the importance of reducing the amount of time spent in SB and engaging in regular physical activity to maintain cardiovascular health.^7^, ^30^ Unfortunately, the recent COVID‐19 pandemic has forced most of the global population to quarantine and spend more time at home, resulting in increased opportunities for SB. In this atypical historical context, it is beneficial to explore the causal relationship between SB and HF, which is the end‐stage manifestation of most heart diseases.^31^

The IVW model has the highest statistical power and is considered the primary standard for MR studies; the MR‐Egger regression model has relatively low statistical power, wider confidence, and insignificant statistical differences.^32^ Based on the comprehensive analysis of the results of the four models used in this study, this MR study indicated a causal relationship between SB and HF, as IVs among an SB‐associated phenotype (TV screen time) showed significant statistical differences in both HF‐associated GWAS datasets. The robustness of the conclusion was demonstrated by sensitivity analysis and is supported by a large number of clinical observational studies; several meta‐analyses have reported that longer TV viewing times are significantly associated with a higher risk of cardiovascular diseases, diabetes, and all‐cause mortality.^33^, ^34^, ^35^ Although another SB‐associated phenotype (driving time) was significantly different in one HF‐associated GWAS data set, statistical significance was not found in the other data set. This indicates that the results here may have insufficient stability, and therefore caution should be exercised when drawing a positive conclusion. It should be noticed that SB of watching TV maybe slightly different from using a computer and driving while the latter must focus attention during the process. However, human on watching may result in not only lower energy expenditure but also with excessive energy (especially snacks) intake or smoking that is harmful to cardiovascular health.^36^, ^37^ Thus, it is recommend limiting TV time to less than 2 h/day to reduce most of the associated adverse health events.^38^

In contrast to SB such as watching TV, physical exercise is an indispensable component of cardiac rehabilitation treatment for patients with HF. Regular aerobic physical exercise is beneficial for reducing the risk of hospitalization and all‐cause mortality as well as improving quality of life.^39^, ^40^ In general, the clinical observations studies surrounding both SB (time of watching TV) and physical exercise help confirm the conclusions of this study.

The results of the mediation analysis indicated that coronary artery disease, hypertension, type 2 diabetes, obesity, and body mass index were major mediators in how SB results in or aggravates HF. Similarly, current mainstream viewpoints suggest that multiple underlying pathways involved in SB leading to HF are associated with exacerbation of primary heart disease and metabolic‐related disorders^41^; the former manifests by accelerating the degree of vascular atherosclerosis, inducing or exacerbating hypertension as well as vascular and endothelial dysfunction, while the latter manifests as obesity, hyperlipidemia, and diabetes. The potential molecular mechanisms include vasoactive mediator imbalance and dysfunction (attenuating nitric oxide and increasing endothelin production), activation of inflammation and oxidative stress, sympathetic activation, and insulin resistance.^42^, ^43^, ^44^ The findings from the European population were also supported by a similar observational study in the Japanese population.^45^

Some limitations of this study should be noted. First, the results of the heterogeneity test suggested certain heterogeneity among the IVs in the study, although the random effects IVW model has been applied to minimize the effect of heterogeneity in MR study as much as possible. Second, models based on different assumptions involved in MR studies have idiosyncratic advantages and disadvantages that may increase the likelihood of obtaining inconsistent or opposite results, meaning that the results should be interpreted with caution. Lastly, the GWAS datasets only focused on the European population, meaning that the findings may not be fully applicable to other ethnicities. Meanwhile, the biological functions of SNPs as IVs and how they aggravate HF deserve further discussion.

## 5 CONCLUSION

This study revealed a causal relationship between SB and HF using an MR methodology, indicating that HF may be prevented by reducing TV screen time. In addition, coronary artery disease, hypertension, and metabolic‐related disorders (type 2 diabetes, obesity, and body mass index) were identified as significant mediators of this causal relationship.

## AUTHOR CONTRIBUTIONS

Xifeng Zheng was responsible for conception and article writing, Manqi Liu was responsible for data mining, Zijun Wu was involved in literature review and Zhen Jia was responsible for scientific supervision. Xifeng Zheng and Manqi Liu contributed equally in the study. All authors reviewed and approved the final manuscript.

## CONFLICT OF INTEREST STATEMENT

The authors declare no conflict of interest.

## Supporting information

Supporting information.

## Data Availability

The genetic variable information of single nucleotide polymorphisms was obtained from the IEU GWAS database (https://gwas.mrcieu.ac.uk/datasets/), a publicly available GWAS summary database.
