# Supplementary material for: Identifying the causal relationship between sedentary behavior and heart failure: Insights from a Mendelian randomization study and mediation analysis
Source: Clin Cardiol. 2023 Aug 29;46(9):1082–9. doi: 10.1002/clc.24101 (PMC10540015; doi:10.1002/clc.24101)
Supplement: Supplementary file 1 — Supporting information. [file CLC-46-1082-s001.docx]

**Supplementary Figure-1 Results of the “leave-one-out” method in the sensitivity analysis.**


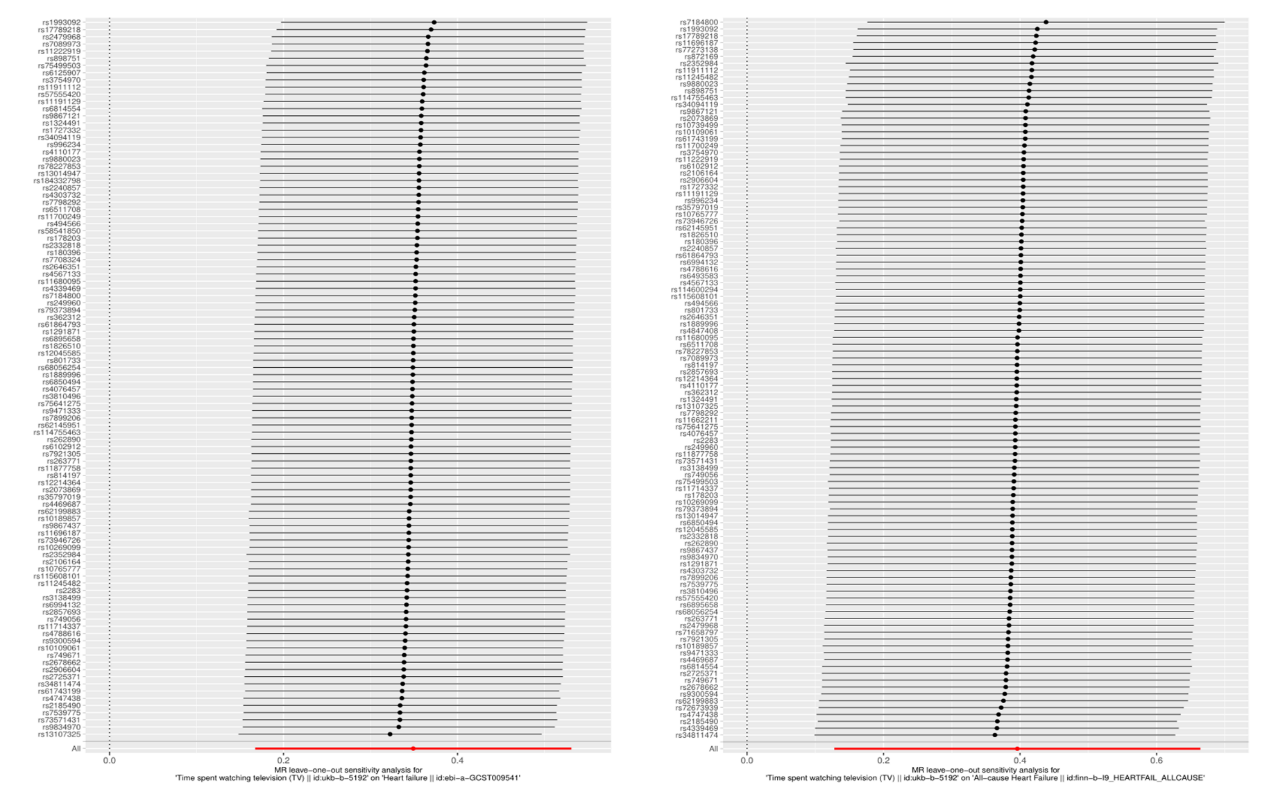


**Note:** The left represents the result of the “leave-one-out” method for the correlation between TV screen time and HF in GWAS dataset ebi-a-GCST009541, and the right represents the resulting relationship between TV screen time and heart failure in GWAS dataset finn-HEARTFAIL. Both results indicated that no specific SNP had a significant effect on the overall result and verified the robustness of the conclusions.
